# Supplementary material for: Analysis of PPARγ Signaling Activity in Psoriasis
Source: Int J Mol Sci. 2021 Aug 10;22(16):8603. doi: 10.3390/ijms22168603 (PMC8395241; doi:10.3390/ijms22168603)
Supplement: Supplementary file 1 [file ijms-22-08603-s001.zip › Supplemental materials_Analysis of PPARg signaling activity in psoriasis/Pathway models/Models images and html files/Interleukin-17 and interleukin-22 signaling in psoriasis/5366.html]

CXCL2


# Protein CXCL2

|  |  |
| --- | --- |
| URN | urn:agi-llid:2920 |
| Total Entities | 0 |
| Connectivity | 2777 |
| Name | CXCL2 |
| Description | chemokine (C-X-C motif) ligand 2 |
| Notes | This antimicrobial gene is part of a chemokine superfamily that encodes secreted proteins involved in immunoregulatory and inflammatory processes. The superfamily is divided into four subfamilies based on the arrangement of the N-terminal cysteine residues of the mature peptide. This chemokine, a member of the CXC subfamily, is expressed at sites of inflammation and may suppress hematopoietic progenitor cell proliferation. [provided by RefSeq, Sep 2014] |
| Primary Cell Localization | Extracellular |
| Class | Ligand |

---

|  |  |
| --- | --- |
| Pathway | CXC Chemokine Receptor Signaling |
|  | Toll-like Receptor Independent Sterile Inflammation |
|  | Neutrophil Chemotaxis |
|  | Vascular Endothelial Cell Activation by Blood Coagulation Factors |
|  | Scavenger Receptor OLR1 in Inflammation Related Endothelial Dysfunction |
|  | Proteins Involved in Myocarditis |
|  | IL17 Signaling in Psoriasis |
|  | Neutrophil Recruitment in Sinovium in Gout |
|  | TLR2 Induced Synovial Fibroblast Activation in Rheumatoid Arthritis |
|  | Proteins Involved in Chronic Bronchitis |
|  | Proteins Involved in Glomerulonephritis |
|  | Endothelial Cell Dysfunction in Glomerulonephritis |
|  | Endothelial Cell Dysfunction in Pyelonephritis |
|  | HMGB1 Expression Targets |
|  | CXCL6 Expression Targets |
|  | CXCL2 Expression Targets |
|  | CXCL3 Expression Targets |
|  | CXCL5 Expression Targets |
|  | CSF2 -> NF-kB Expression Targets |
|  | F2 -> AP-1/CREB/ELK/SRF/SP1 Expression Targets |
|  | F2 -> STAT1/NF-kB Expression Targets |
|  | HGF -> AP-1/CREB/ELK/SRF/MYC Expression Targets |
|  | TAM Receptor Signaling Activation in Cancer |
|  | Fibronectin Expression Targets |
|  | ICAM1 Expression Targets |
|  | IL15 Expression Targets |
|  | Proteins/Ions Transporters -> Expression Targets in Lymphoid System and Blood |
|  | Elevated Receptors -> Expression Targets in Lymphoid System and Blood |
|  | GPCRs Family -> Expression Targets in Lymphoid System and Blood |
|  | TNF Receptors -> Expression Targets in Lymphoid System and Blood |
|  | P2RX7 -> Expression Targets in Nerve Tissue |
|  | PDGF -> AP-1/CREB/CREBBP/MYC Expression Targets |
|  | AGT -> CREB Expression Targets |
|  | FASLG Expression Targets |
|  | TNF -> NF-kB Expression Targets |
|  | TNF -> CREB Expression Targets |
|  | TLR5 Expression Targets |
|  | TLR1 -> 2/6 Expression Targets |
|  | IL1B Expression Targets |
|  | TLR4 -> NF-kB/IRF Expression Targets |
|  | IL1A Expression Targets |
|  | ROS in Triggering Vascular Inflammation |
|  | pathway 3 |
|  | e |
|  | pathway 4 |
|  | New Pathway (2) |
|  | pprarg neg, uknown targets, ps-positive |
|  | comon ps\_pos, pprarg\_neg targets |
|  | New Pathway (4) |
|  | 2\_Interleukin-17 and interleukin-22 signaling in psoriasis |
|  | 2\_The role of endothelial cells in pyelonephritis |
|  | 2\_Oxidized LDL causes endothelial dysfunction in atherosclerosis |
|  | drug-target moltransport |
|  | Interleukin-17 and interleukin-22 signaling in psoriasis |

---

|  |  |
| --- | --- |
| MedScan ID | 2920 |

---

|  |  |
| --- | --- |
| LocusLink ID | 2920 |
|  | 14825 |
|  | 81503 |

---

|  |  |
| --- | --- |
| Alias | secretory protein N51 |
|  | GRO2 oncogene |
|  | CXC ligand 2 |
|  | small inducible cytokine subfamily member II |
|  | MI2A\_HUMAN |
|  | MIP2-alpha |
|  | C-X-C motif chemokine 2 |
|  | chemokine CXCL2 |
|  | macrophage-inhibitory protein-2 |
|  | CXCL2 |
|  | platelet-derived growth factor-inducible protein KC |
|  | CINC-2a |
|  | GROB |
|  | macrophage inflammatory protein-2-alpha |
|  | growth regulated protein beta |
|  | melanoma growth stimulatory activity beta |
|  | chemokine (C-X-C motif) ligand 2 |
|  | MGSA beta |
|  | Scyb |
|  | macrophage-inhibitory protein-2 alpha |
|  | MIP2 |
|  | MGSA-b |
|  | small inducible cytokine B2 |
|  | Gro-beta |
|  | chemokine (C-X-C motif) ligand II |
|  | small inducible cytokine subfamily member 2 |
|  | LOC133087 |
|  | CXCL2 chemokine |
|  | chemokine CXC ligand 2 |
|  | SCYB2 |
|  | GRO2 |
|  | CXC chemokine ligand 2 |
|  | growth-regulated protein beta |
|  | MIP2A |
|  | MIP-2a |
|  | macrophage inflammatory protein 2-alpha |
|  | KC |
|  | Fsp |
|  | N51 |
|  | gro |
|  | Gro1 |
|  | Mgsa |
|  | Scyb1 |
|  | C-X-C motif chemokine 1 |
|  | GRO1 oncogene |
|  | KC/GR)-alpha |
|  | KC/GRO-alpha |
|  | alpha-chemokine |
|  | growth-regulated alpha protein |
|  | CINC-1 |
|  | chemokine (C-X-C motif) ligand 1 (melanoma growth stimulating activity, alpha) |
|  | cytokine-induced neutrophil chemoattractant 1 |

---

|  |  |
| --- | --- |
| GO ID | 0008009 |
|  | 0007186 |
|  | 0061844 |
|  | 0071222 |
|  | 0070098 |
|  | 0006935 |
|  | 0019221 |
|  | 0006955 |
|  | 0006954 |
|  | 0030595 |
|  | 0030593 |
|  | 0002237 |
|  | 0005576 |
|  | 0005615 |
|  | 0005125 |
|  | 0008083 |
|  | 0002526 |
|  | 0006952 |
|  | 0007204 |
|  | 1902035 |
|  | 0070965 |
|  | 0043268 |
|  | 0010765 |
|  | 0032930 |
|  | 0060326 |
|  | 0010469 |
|  | 0001975 |
|  | 0032355 |
|  | 0010332 |
|  | 0051384 |
|  | 0009408 |
|  | 0032496 |
|  | 0005623 |
|  | 0045236 |
|  | 0090023 |
|  | 0042127 |
|  | 0005829 |
|  | 0002690 |

---

|  |  |
| --- | --- |
| KEGG ID | hsa:2920 |
|  | mmu:14825 |
|  | rno:81503 |

---

|  |  |
| --- | --- |
| Organism | Homo sapiens {Organism urn:agi-taxid:9606} |
|  | Mus musculus {Organism urn:agi-taxid:10090} |
|  | Rattus norvegicus {Organism urn:agi-taxid:10116} |
|  | Homo sapiens |
|  | Mus musculus |
|  | Rattus norvegicus |

---

|  |  |
| --- | --- |
| Mouse chromosome position | 5 44.78 cM |

---

|  |  |
| --- | --- |
| OMIM ID | 139110 |

---

|  |  |
| --- | --- |
| Rat chromosome position | 14p22 |

---

|  |  |
| --- | --- |
| Hugo ID | 4603 |
|  | HGNC:4603 |

---

|  |  |
| --- | --- |
| Human chromosome position | 4q13.3 |
|  | 4q21 |

---

|  |  |
| --- | --- |
| Swiss-Prot Accession | A0A024RDD9 |
|  | P19875 |
|  | P19875.1 |
|  | A2RTH0 |
|  | P12850 |
|  | P12850.1 |
|  | P14095 |
|  | P14095.2 |
|  | Q6FGD6 |
|  | Q9UPB8 |

---

|  |  |
| --- | --- |
| PIR ID | JH0281 |
|  | A32954 |
|  | JN0572 |

---

|  |  |
| --- | --- |
| GenBank ID | NC\_000004 |
|  | NM\_002089 |
|  | NP\_002080 |
|  | AC093677 |
|  | CH471057 |
|  | EAX05700 |
|  | EAX05701 |
|  | M65006 |
|  | AAP13104 |
|  | U03019 |
|  | AAA03453 |
|  | AF043340 |
|  | AAC03540 |
|  | AK308504 |
|  | BC015753 |
|  | AAH15753 |
|  | BC053653 |
|  | BX334969 |
|  | CR542171 |
|  | CAG46968 |
|  | M36820 |
|  | AAA63183 |
|  | M57731 |
|  | AAA63182 |
|  | X53799 |
|  | CAA37808 |
|  | P19875 |
|  | NC\_000071 |
|  | NM\_008176 |
|  | NP\_032202 |
|  | AC157938 |
|  | AH003603 |
|  | AAB03376 |
|  | CH466617 |
|  | EDL05301 |
|  | KX832958 |
|  | S79767 |
|  | AAP32036 |
|  | AF352782 |
|  | AK140312 |
|  | BC037997 |
|  | AAH37997 |
|  | BC132502 |
|  | AAI32503 |
|  | BC132504 |
|  | AAI32505 |
|  | BQ031102 |
|  | BY335927 |
|  | J04596 |
|  | AAA40131 |
|  | P12850 |
|  | NC\_005113 |
|  | NM\_030845 |
|  | NP\_110472 |
|  | AC\_000082 |
|  | AAHX01078373 |
|  | AC108576 |
|  | CH474060 |
|  | EDL88575 |
|  | D11445 |
|  | BAA02009 |
|  | D11444 |
|  | BAA02008 |
|  | M86536 |
|  | AAA42053 |
|  | P14095 |
|  | NC\_018915 |
|  | AMYH02009474 |
|  | AC\_000027 |
|  | AAHY01046755 |
|  | U20527 |
|  | U20634 |
|  | AC\_000136 |
|  | ABBA01022994 |

---

|  |  |
| --- | --- |
| Swiss-Prot ID | CXCL2\_HUMAN |
|  | GROA\_MOUSE |
|  | GROA\_RAT |

---

|  |  |
| --- | --- |
| Cell Localization | Secreted |

---

|  |  |
| --- | --- |
| Ensembl ID | ENSG00000081041 |
|  | ENSP00000427279.1 |
|  | ENST00000508487.3 |
|  | ENSMUSG00000029380 |
|  | ENSMUSP00000031327.8 |
|  | ENSMUST00000031327.8 |
|  | ENSRNOG00000002802 |
|  | ENSRNOP00000003778.2 |
|  | ENSRNOT00000003778.3 |
|  | ENSP00000427279 |
|  | ENST00000508487 |
|  | ENSMUSP00000031327 |
|  | ENSMUST00000031327 |
|  | ENSRNOP00000003778 |
|  | ENSRNOT00000003778 |

---

|  |  |
| --- | --- |
| MGI ID | MGI:108068 |

---

|  |  |
| --- | --- |
| RGD ID | 619869 |

---

|  |  |
| --- | --- |
| Unigene ID | Hs.75765 |
|  | Mm.21013 |
|  | Rn.10907 |

---

|  |  |
| --- | --- |
| Homologene ID | 105490 |

---

|  |  |
| --- | --- |
| Shape | Rhomb |

---
